# Case Report of Spontaneous Thyroid Hemorrhage Following LMA Insertion

**DOI:** 10.21980/J8XP8W

**Published:** 2020-07-15

**Authors:** Gregory Podolej, Gary Bhagat

**Affiliations:** * University of Illinois College of Medicine – Peoria, Department of Emergency Medicine, Peoria, IL

## Abstract

A case of thyroid hemorrhage following laryngeal mask airway (LMA) intubation is described. A 42-year-old female presented to the emergency department (ED) with left neck pain after same day surgery with LMA intubation and was found to have a thyroid hemorrhage on imaging. The patient was ultimately discharged in stable condition with no acute interventions performed in the ED or by otorhinolaryngology (ENT). Airway implications and possible interventions based on isolated case reports are reviewed and discussed.

**Topics:**

Thyroid hemorrhage, LMA complications.

**Figure f1-jetem-5-3-v10:**
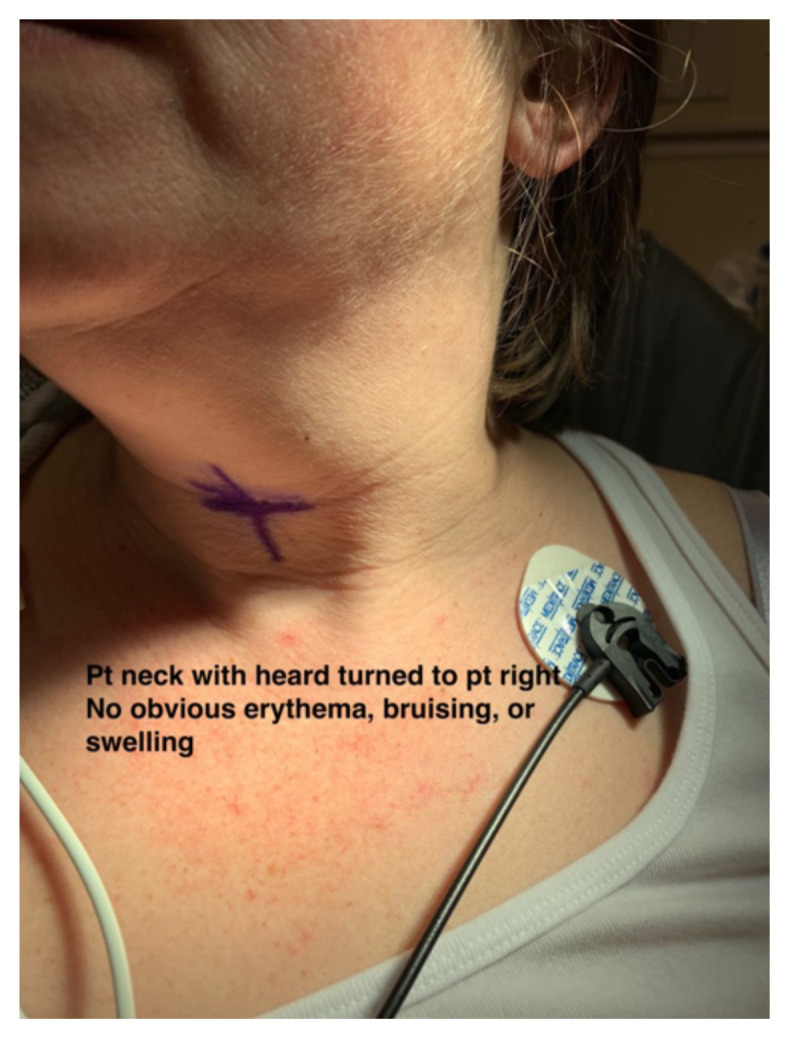


**Figure f2-jetem-5-3-v10:**
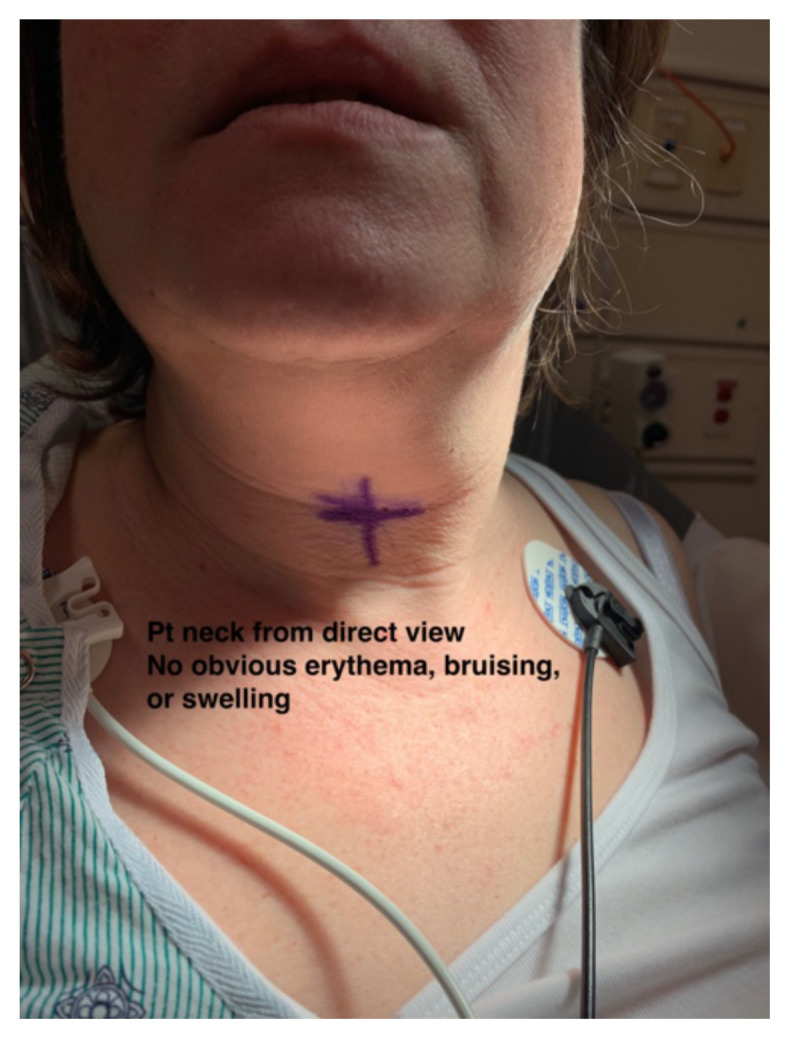


**Figure f3-jetem-5-3-v10:**
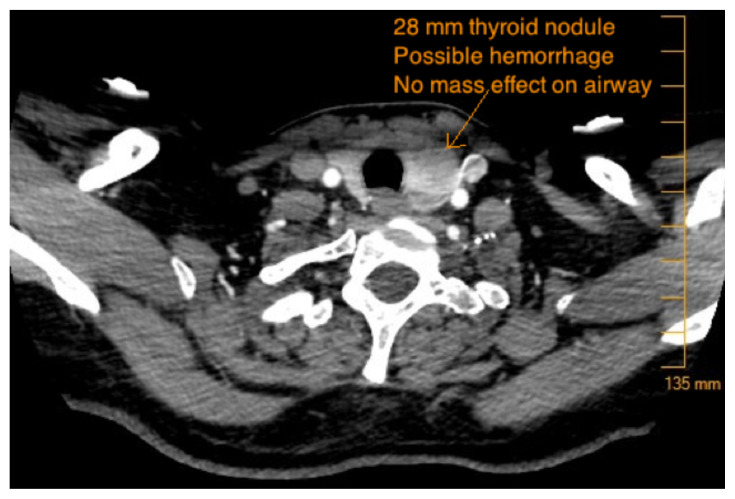
Axial CTA Video Link: https://youtu.be/Hxoxi8FHvmk [Fig f1-jetem-5-3-v10][Fig f2-jetem-5-3-v10][Fig f3-jetem-5-3-v10]Sagital CTA Video Link: https://youtu.be/Xx_HeC6N6Es Coronal CTA Video Link: https://youtu.be/Vuophy3ublE Axial CT Soft Tissue Video Link: https://youtu.be/K1mMRIlBVqg

## Introduction

The thyroid gland is a highly vascularized bi-lobed gland whose small isthmus overlays the trachea at the level of the second and third tracheal rings.^1^,^2^ Preexisting thyroid conditions such as goiters, cysts, or nodules can lead to increased baseline risk of spontaneous hemorrhage.^3^,^4^ External blunt neck trauma has been seen in isolated cases to cause thyroid hemorrhage. 5–7 Even less common is endotracheal (ET) intubation leading to thyroid hemorrhage.^1^ Regardless of the cause, thyroid hemorrhages and hematomas present unique airway management challenges that are not typically encountered.^3^–^7^

We present the case of traumatic hemorrhage into thyroid cyst following a laryngeal mask airway (LMA) insertion.

## Presenting concerns and clinical findings

A 42-year-old Caucasian female with no significant past medical history presented to the emergency department (ED) for left-sided neck pain and swelling after having a scheduled outpatient hysteroscopy with surgical dilatation and curettage earlier that morning. During her procedure, the patient received generalized anesthesia consisting of propofol, lidocaine, dexamethasone, ondansetron, fentanyl and ketorolac. The patient had an LMA placed for airway management while under general anesthesia. The patient was discharged in a stable condition after her procedure. Following discharge, the patient began having left-sided neck pain and swelling. She contacted her surgeon who performed the procedure and was referred to the ED.

On initial evaluation, the patient endorsed left-sided neck pain, fullness and decreased range of motion at the neck. The patient denied any sore throat, difficulty swallowing, hemoptysis, nausea or vomiting. On examination the patient had stable vital signs, BP 132/62 mmHg, pulse 73 beats per minute, temperature 98.9ºF, respiratory rate 22 breaths per minute, pulse oximetry 97% on room air. The patient’s neck was examined and no obvious deformity, swelling, or bruising could be appreciated to explain her symptoms (see photographs).

## Patient Course

The patient had normal bloodwork. Based on imaging findings, ENT was consulted who recommended observation and thyroid ultrasound. Thyroid ultrasound revealed 3.4cm left thyroid nodule with normal sonographic appearance of the thyroid parenchyma. The patient was admitted, observed, and discharged the next day with follow up after ENT evaluation was performed the next day. Otorhinolaryngology believed it was a traumatic intracapsular hemorrhage within the thyroid nodule that would self-resolve. Patient ultimately had the thyroid nodule removed.

## Significant findings

Two photographs of patients neck, both showcasing no obvious erythema, bruising, or swelling which is noteworthy because there is potential for airway compromise but there was nothing visible to indicate that on exam.

CTA of neck showing thyroid nodule and potential thyroid hemorrhage (outlined in orange) on the left without evidence of airway compromise at the time of CT scan. Official read by attending radiologist states there is a “heterogeneous left thyroid nodule measuring 3 cm. Findings are suggestive of multinodular goiter with possible acute hemorrhage. Adjacent tract of soft tissue stranding in the anterior left neck with mild adjacent fascial thickening. This could represent small amount of hemorrhage or could be inflammatory.”

## Discussion

The thyroid gland is a bi-lobed endocrine gland found in the anterior neck. It is located deep to the overlying muscles, within the visceral compartment of the neck, and lays against the trachea.^1^,^2^ It is a highly vascular structure receiving its arterial supply from the superior and inferior thyroid arteries and their branches. The superior thyroid artery branches off the external carotid artery, and the inferior thyroid artery branches off of the thyrocervical trunk, which itself is a branch from the subclavian artery.^1^,^2^ An anatomic variant known as the thyroid ima artery exists in up to 10% of the population, and when present runs its course along the anterior aspect of the trachea and supplies blood to the anterior thyroid as well as the isthmus.^1^,^2^ The venous drainage system for the thyroid consists of the superior, middle, and inferior thyroid veins bilaterally that drain into either the subclavian or brachiocephalic veins. The superior thyroid veins will primarily drain the areas supplied by the superior thyroid arteries, and the middle and inferior thyroid veins will drain the remainder of the thyroid.^1^,^2^

Thyroid hemorrhages and hematomas, whether arising spontaneously or through trauma, are not common occurrences.^3^–^9^ Traumatic hemorrhage within the thyroid following intubation or other airway procedure is a rare occurrence with few documented cases.^3^,^4^ No exact mechanism is known for the cause of these bleeds; however, it has been noted that the presence of a pre-existing thyroid nodule or goiter increases the risk of hemorrhage or hematoma formation.^5^,^10^ One possible mechanism that has been considered involves arteriovenous shunting within the nodules. Thyroid nodules may contain abnormal vasculature with weaker venous systems, and if the nodule experiences increased pressure from exertion or trauma, this could lead to bleeding within the nodule.^10^ Given the anatomical positioning of the thyroid in relation to the airway, hemorrhage hematoma within the thyroid or surrounding the thyroid has the potential to lead to airway compromise, respiratory distress, and possibly cardiac arrest.^7^,^9^,^10^ Furthermore, the presentation may make standard ET intubation or emergent cricothyrotomy difficult, and techniques utilizing airway adjuncts such as bougies may need to be first line.^5^–^10^ Case reports in the setting of external neck trauma have reported that patients may have delayed presentations of up to or greater than 24 hours from initial presentation.^5^,^7^ Patients with signs of thyroid hemorrhage should be closely observed with airway equipment available and may require admission for observation.^5^,^9^

This case report aims to highlight the fact that thyroid hemorrhage can be a rare but potentially life-threatening complication of LMA ventilation. Thyroid hemorrhage is not often the preeminent diagnosis for a patient presenting with neck discomfort, but this case report posits that it should be considered more frequently, particularly for patients who present with neck discomfort post-procedure.

## Supplementary Information




















