# Supplementary figures and images for: Case Report of Spontaneous Thyroid Hemorrhage Following LMA Insertion
Source: J Educ Teach Emerg Med. 2020 Jul 15;5(3):V10–3. doi: 10.21980/J8XP8W (PMC10332558; doi:10.21980/J8XP8W)

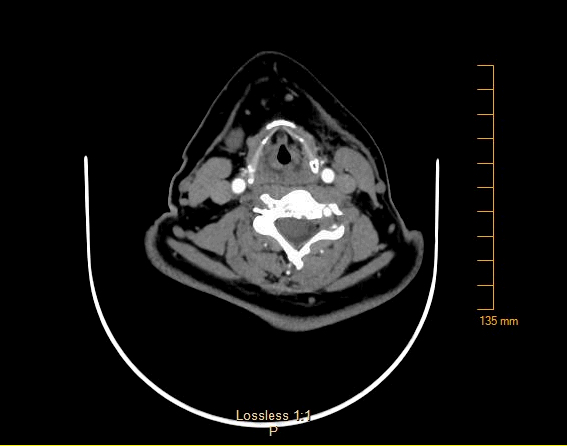

Supplement: Supplementary file 1 [file jetem-5-3-v10-supp1.jpg]

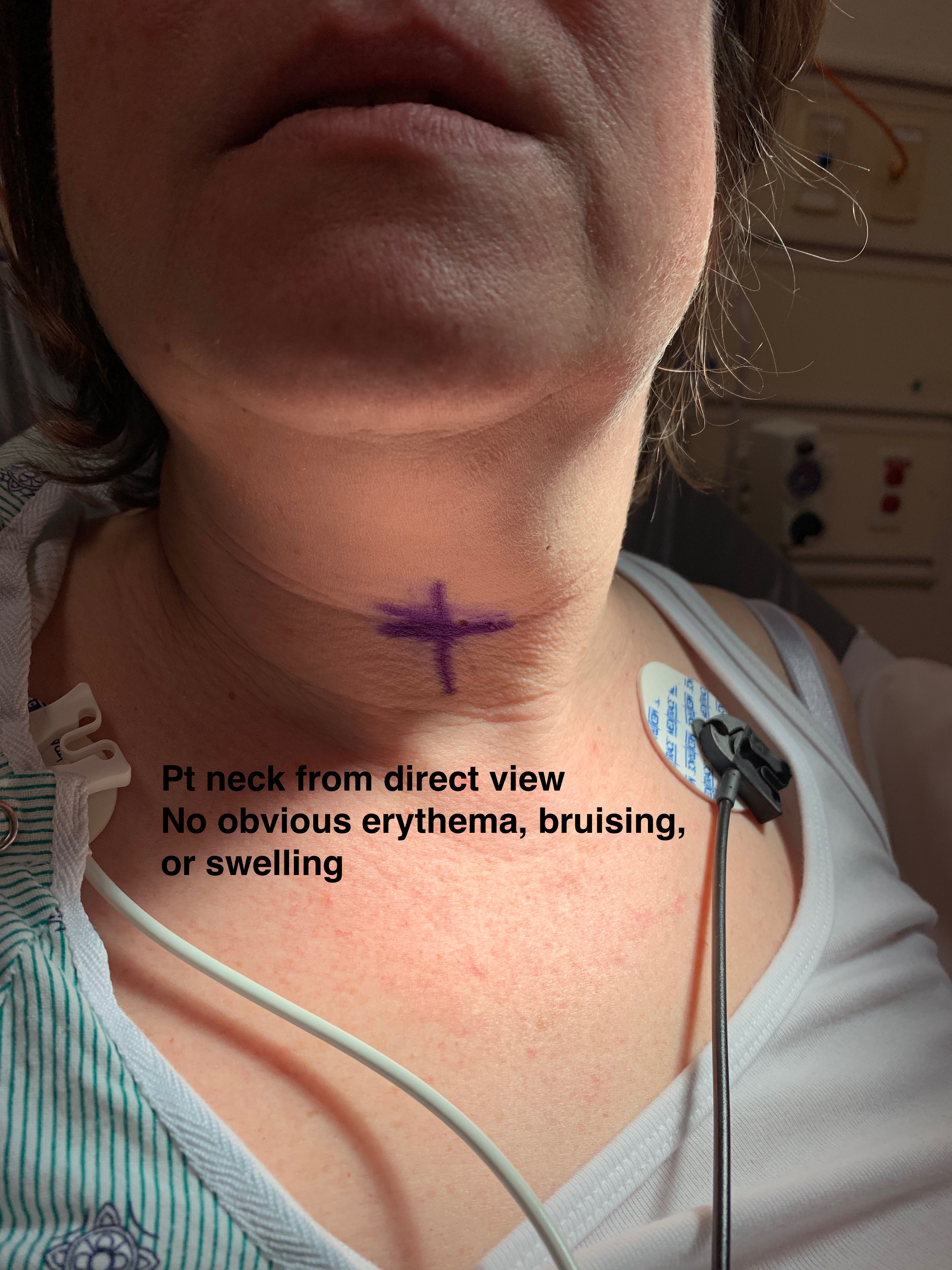

Supplement: Supplementary file 6 [file jetem-5-3-v10-supp6.jpg]

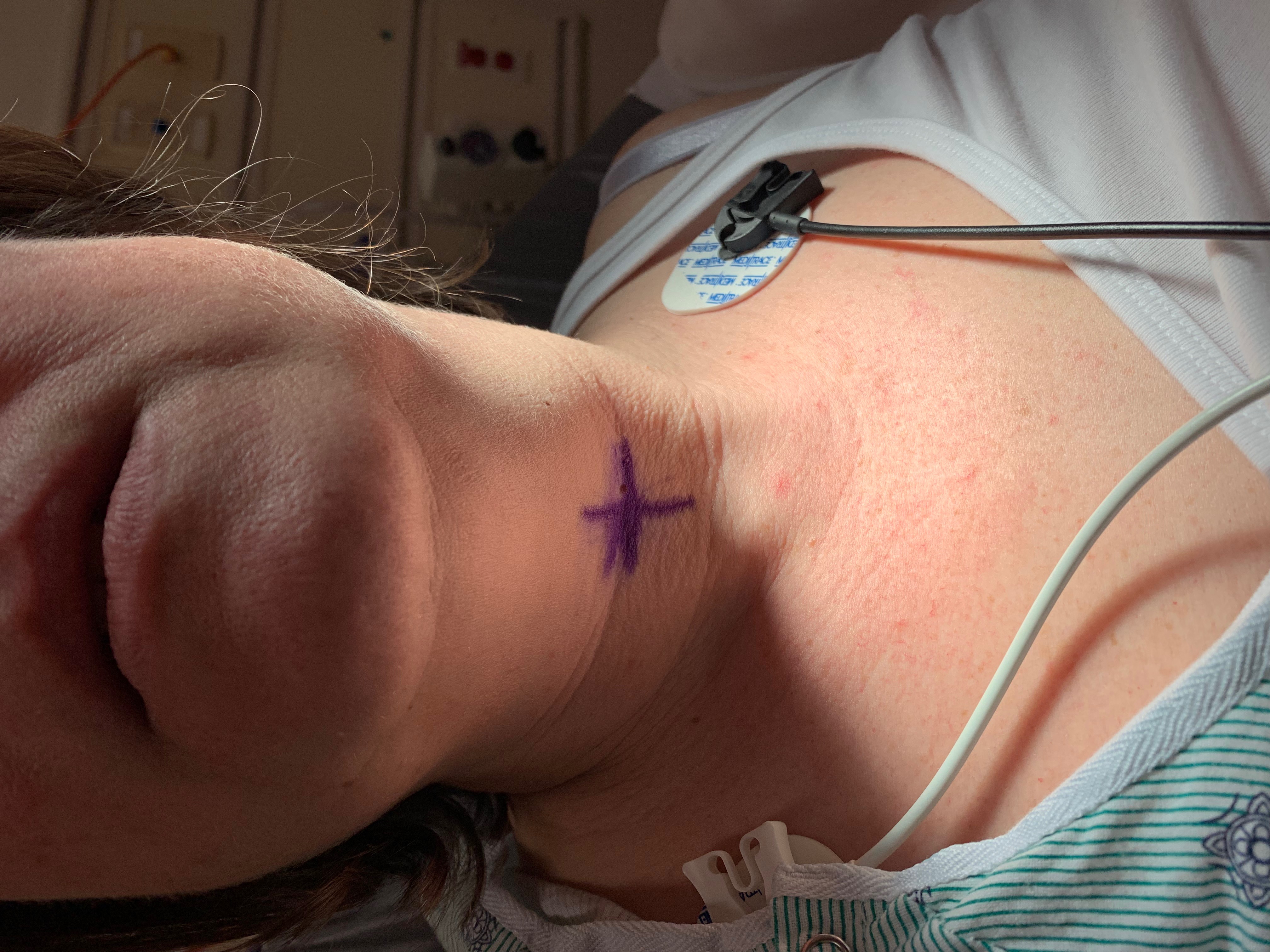

Supplement: Supplementary file 7 [file jetem-5-3-v10-supp7.jpg]

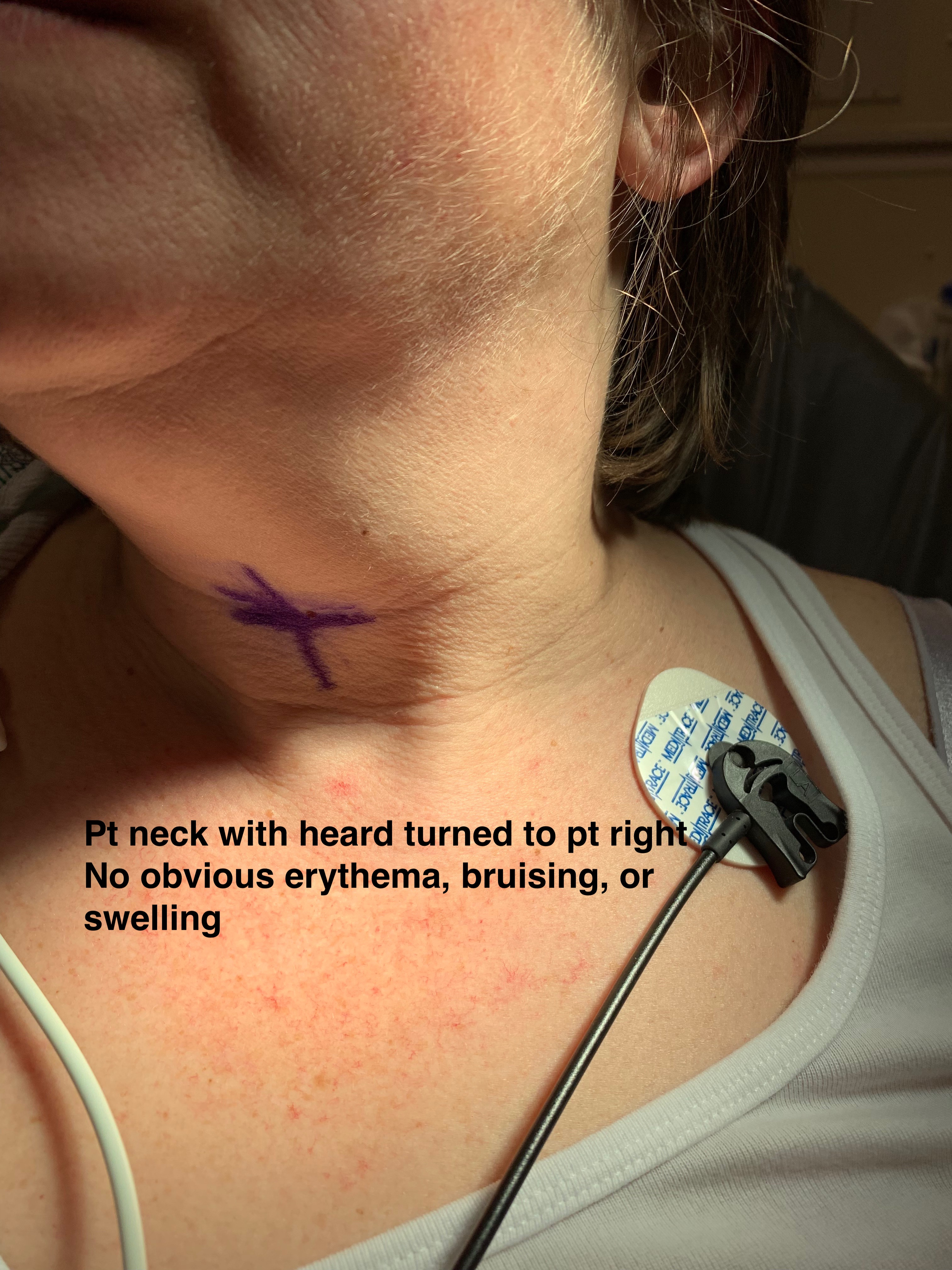

Supplement: Supplementary file 8 [file jetem-5-3-v10-supp8.jpg]

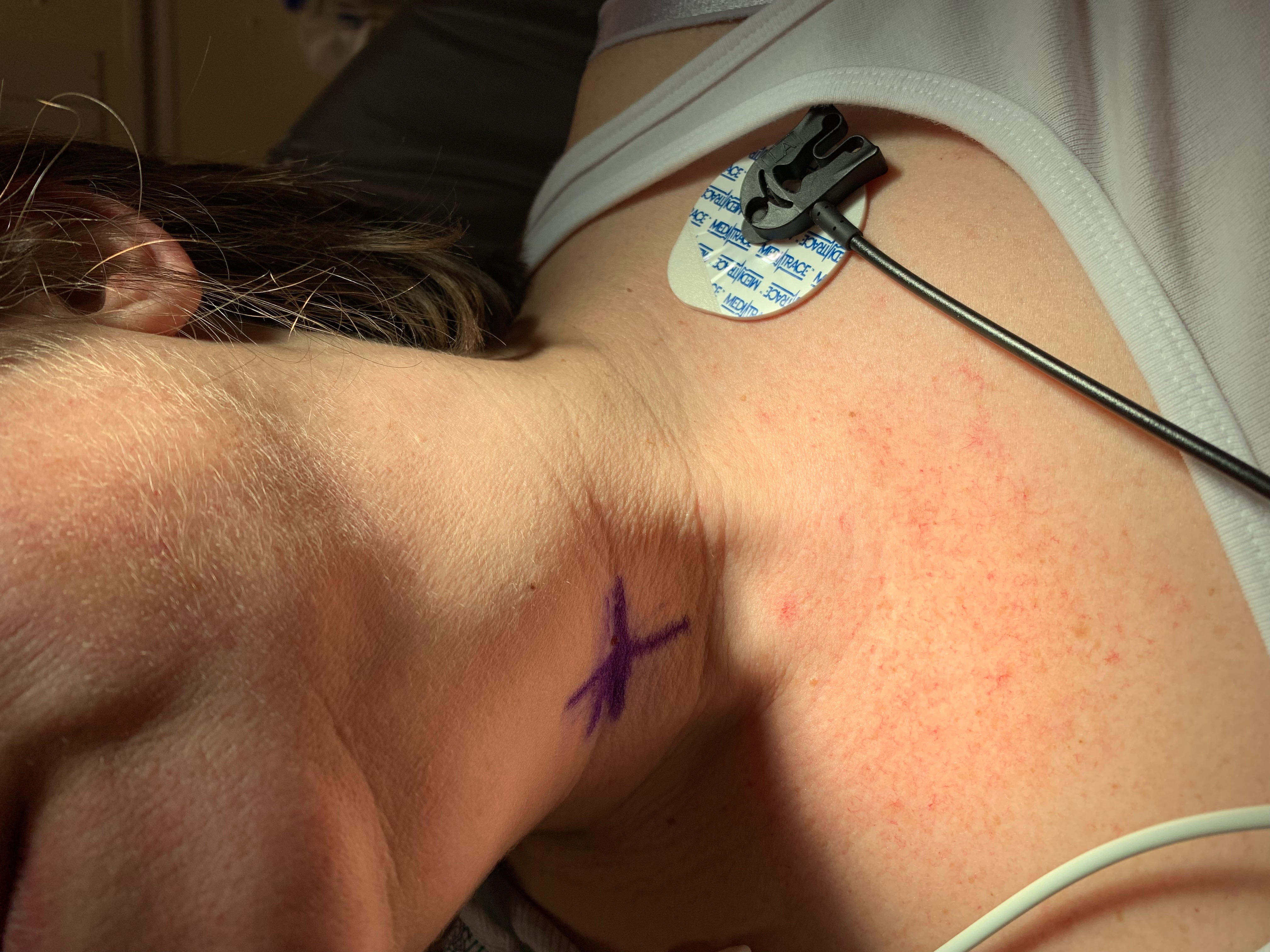

Supplement: Supplementary file 9 [file jetem-5-3-v10-supp9.jpg]

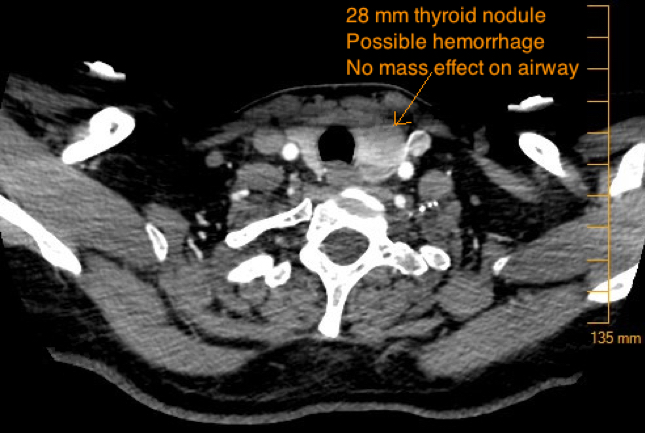

Supplement: Supplementary file 10 [file jetem-5-3-v10-supp10.jpg]
